# Therapeutical attitudes in tooth supported overdentures with ball attachements. Case report

**Published:** 2014

**Authors:** AM Țâncu, M Melescanu Imre, CT Preoteasa, E Preoteasa

**Affiliations:** * Department of Prosthodontics, Faculty of Dental Medicine, “Carol Davila” University of Medicine and Pharmacy; ** Department of Oral Diagnosis, Ergonomics, Scientific Research Methodology, Faculty of Dental Medicine, “Carol Davila” University of Medicine and Pharmacy

**Keywords:** overdenture, natural teeth, insufficient clinical examination

## Abstract

Tooth supported overdenture with ball attachments has a number of advantages in prosthetics, but presents some difficulties as well, which sometimes make impossible the use of these anchoring systems; these difficulties should be well known. In this regard we present a suggestive case. It is the case of a patient, aged 57, who came for treatment, suffering from subtotal maxillary and mandibular edentulism (present 11 and 21, respectively 33 and 43), previously having an overdenture prosthesis on natural teeth, with special systems – ball attachment type, dissatisfied with the treatment (due to repeated fractures and functional intolerance to dentures). Clinical examination revealed an increase of the vertical dimension of occlusion and reduced prosthetic space at a correct, functional DVO value, aspects that were explaining the patient’s reported complaints. As a therapeutic approach, having into consideration the balance conditions that were favorable for complete dentures and the large ball attachments volume, which did not allow keeping them at a functional DVO at any of the jaws, and the relatively young age of the patient, it was decided to remove the ball attachments and to keep the teeth for a simple overdenture, both to the maxillar and the mandible, over coronary reduced teeth, enough to allow the denture thickness at a functional DVO. Good end result of prosthetics, with stable, functional dentures, which were well tolerated and offered satisfaction to the patient, have led to an increased quality of life.

## Introduction

Tooth supported overdenture is a partial or complete denture, which is based on one or more teeth or tooth roots. Through their presence in the prosthesis they reduce bone resorption, compared to the conventional complete denture. „To keep a few teeth and use them or their roots for a tooth or root-supported overdenture substantially reduces bone loss.” [**1**,**2**] 

This type of prosthetic treatment offers many advantages over conventional dentures, being more stable, functional, patients having a much better mastication. Keeping even a small number of teeth [**1**,**2**], as roots kept under dentures, has a strong psychological effect on some patients. All these aspects indicate them frequently in patients within current practice, in the presence of the last teeth, that can be kept under dentures.

Interest on tooth supported overdenture is demonstrated by the numerous studies presented in specialized publications. Thus, consulting some of the international databases, using the keywords ”**tooth supported overdenture**”, resulted that in **PubMed** between 2000 –2014 there have been published on this topic 275 articles with or without full text, in **Science Direct**, 1005 articles, with and without full text, in **Ebsco Dentistry & Oral Sciences** 20 full-text articles, and in **Wiley Outline Library**, 1039 articles, with and without full text, which refer to the searched keywords.

## Case presentation

Patient S.A., aged 57, suffering of **subtotal maxillary and mandibular edentulism**, came to the Department of Complete Prosthetics, with 2 mandibular overdentures, both fractured after about 2-3 months after application, and a maxillary overdenture, made about 4 months ago, saying she was totally dissatisfied with them and asking for their restoration. Clinical evaluation of the patient, starting from questioning, from which we learned the history of prosthetics and her discontents, completed with an objective physical exam of the patient and the prosthetics, revealed a number of incorrect aspects. We noted in this regard aspects related to the mandible overdenture fracture shortly after its application, denture reconstruction, followed by the same repeated fracturing. At the same time the patient declared that she felt a discomfort caused by dentures and had difficulties wearing them.

DVO evaluation highlighted an increase of its value well above the functional one (over 7 mm). In determining DVO, based on functional methods, we found that the vertical prosthetic space was reduced, insufficient to store the special systems into the two jaws, not even to one, not being able to provide prosthesis base thickness, which ensures the prosthetic parts resistance and prevents their fracture. Under these conditions, prosthetic rehabilitation by simple tooth supported overdenture without the use of ball attachments, by making dentures thoroughly in all clinical and technical stages resulted in obtaining dentures that were stable, functional, well accepted and tolerated by the patient.[**6**]

**Fig. 1:**
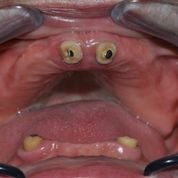
Maxillary and mandibulary prosthetic field

**Clinical evaluation** consisted in examining the prosthetic field, the remaining teeth (11 and 21, respectively 33 and 43), where retention systems were applied (ball attachment type) as well as present overdentures, and respectively the causes of their fracture (**Fig. 1**)

**Fig. 2:**
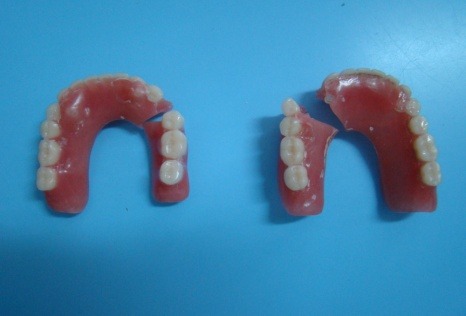
The 2 mandibulary overdentures

**Fig. 3:**
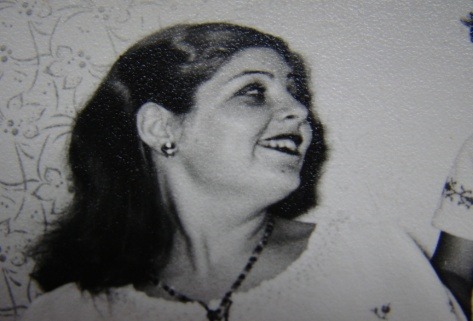
Patient’s S.A. foto from the youth period

There were also conducted study models, imagistic evaluation (through retroalveolar radiography for the remaining teeth), foto`s from the past (**Fig. 3**) and prosthesis evaluation, intra and extraoral.(**Fig. 2**)

Retroalveolar radiographs of remaining teeth revealed the correctness of endodontic treatments and a proper implantation of involved teeth.

Overdentures examination has revealed the incorrect implementation of mandibular frontal teeth, the lateral maxillary and mandibular teeth, and base expansion (maxillary short within distal area) and marginal compliance (especially in the central lingual area).

Dentures intolerance declared by patient, but also intraoral examination of overdentures have highlighted an incorrectly established DVO, being much larger. Besides, the aesthetic appearance of the patient also betrayed the DVO overrating: high teeth visibility, difficulty when closing lips with wry facies look. To establish the correct vertical relation between the two jaws and to determine DVO we used several methods, taking measurements in the lower part of the face, with and without previous dentures, as DVR and DVO values, and especially using functional methods (based on rest and phonation). Thus we found out that, at the old dentures, the occlusion’s vertical dimension of the lower part of the face was increased by more than 7 mm, which explains the repeated fracture of the mandibular denture, after a short period from the application point.[**9**] By increasing DVO an attempt was made to obtain vertical prosthetic space required when applying the ball attachment systems. Following the enlarged DVO and the incorrect teeth implementation of the two artificial arches, there have appeared negative functional aspects, with fractures and difficulties in adapting to dentures.

Evaluation of prosthetic field, regarding the prosthetic conditions showed good balance conditions for complete dentures: tall and wide ridges to the maxilla and average to the mandible, left tuberosity retention in transversal direction, palatal arch with average depth. Corroborating these issues with reduced prosthetic space due to ball attachment type systems, massive systems, led us to the **conclusion** that this type of prostheses anchoring systems on teeth could not be used, their volume being too large in relation to the vertical prosthetic space, present between the two ridges at functional DVO.

**Treatment plan**

After the evaluation and corroboration of all clinical data, we proceeded with the removal of the 4 ball attachment systems present on teeth and we kept the teeth, as roots under the dentures, with obturations from composite material, applied in cavities prepared when entering the canals.[**4**] Afterwards, we continued with all clinical and technical stages specific to tooth supported overdentures. We paid extra attention to the phase when we determined the intermaxillary relations and to the stage when we implemented the teeth, in order to avoid previous mistakes.[**5**] In order to implement the teeth, we asked the patient for photos when she was young, photos that could give us additional data. The patient has provided us with the photos and we could perform an assessment regarding the visibility of the anterior maxillary teeth, shape, size and their position. Previous dentures and models were checked, comparatively, regarding DVO and teeth visibility, and after manufacturing the overdentures in the laboratory, they were applied into the buccal cavity, the patient being satisfied with the final result. So, the dentures were able to be worn, with a good balance and allowed a smooth running of functional activities, being well tolerated.

**Fig. 4:**
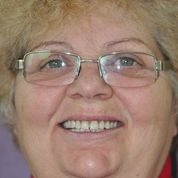
Patient S.A. with the new overdentures

Overdenture, as correct made dentures, on natural teeth, without special systems, had long -term good results, so after regular checks and verification, for 2 years, every 6 months, the patient declared herself satisfied with the dentures. (**Fig. 4**)

## Discussion and conclusions

Overdenture concept, developed as a simple and economical alternative to extend the presence of the last remaining teeth on the arch, especially to the mandible, and to reduce mandibular bone resorption, remains a viable treatment alternative for subtotal edentulism,especially to the mandible.[**11**]

Incomplete assessment of the clinical situation, especially of the vertical interarch space, ignorance or disregard of the principles and clinical and technical stages, lead to serious errors in prosthetics (by overrating DVO, improper teeth implementation). These are followed by functional and prosthetic complications, such as the inability to chew, phonation, dentures intolerance and increased risk of repeated fractures, with a negative impact on the patient’s social relationships and quality of life.

Ball attachment systems are known due to their benefits in overdenture’s retention, but it is also known the aspect related to their important volume, compared to other systems (magnetic). As a result, their usage in case of a reduced vertical prosthetic space, can lead to inadequate prosthetic treatment, accompanied by unpleasant functional and biomechanical consequences, dentures becoming intolerable to the patient.

The patient’s evaluation, given the favorable balance conditions of the complete dentures and the large volume of ball attachments, which, with functional DVO, did not allow keeping them to none of the jaws, the relatively young age of the patient, led to a therapeutic attitude to remove the ball attachments and keep the teeth for a simple overdenture, both to maxilla and mandible, which allowed the denture thickness to a functional DVO, able to reduce the risk of the denture’s fracture. The good end result of prosthetics, through functional balance of dentures, well tolerated by patient, that gave her satisfaction, led to an increased quality of life.[**7**,**10**]

Tooth supported overdenture continues to be an accepted treatment method for both practitioners and patients, with good results, this treatment’s principles being adopted in overdenture on implants and mini implants, that nowadays are usual therapies worldwide.[**3**,**8**]
